# The relationship between internet addiction and lifestyle among high school students: A cross sectional in the west of Iran

**DOI:** 10.1371/journal.pone.0308333

**Published:** 2024-09-06

**Authors:** Azad Shokri, Asra Mohamadi, Donya Mohammadi, Mahana Moradi, Shahram Sadeghi, Hassan Mahmoodi, Kaveh Qaderi Bagajan

**Affiliations:** 1 Social Determinants of Health Research Center, Research Institute for Health Development, Kurdistan University of Medical Sciences, Sanandaj, Iran; 2 Student Research Committee, Kurdistan University of Medical Sciences, Sanandaj, Iran; 3 Spiritual Health Research Center, Research Institute for Health Development, Kurdistan University of Medical Sciences, Sanandaj, Iran; 4 Department of Clinical and Health Psychology, Shahid Beheshti University, Tehran, Iran; Group for Technical Assistance / Asian College for Advance Studies, Purbanchal University, NEPAL

## Abstract

Excessive internet usage can precipitate internet addiction (IA), negatively impacting lifestyle behaviors, especially during adolescence. These warrants investigating associations between IA and lifestyle factors. To examine the relationship between IA and health-promoting lifestyle dimensions among Iranian high school students. A cross-sectional study was conducted among 407 students from Sanandaj, Iran selected via cluster sampling. IA was assessed using the Young IA Test. Lifestyle was measured via the Health-Promoting Lifestyle Profile questionnaire. Regression analyses evaluated associations while adjusting for covariates. Approximately 25% of participants were at risk of internet addiction. Prevalence variations across groups lacked significance. Non-addicted students had significantly higher overall healthy lifestyle scores (p<0.05). Spiritual growth, health responsibility and nutrition scores significantly differed between addicted and non-addicted students. Regression analyses revealed a statistically significant negative correlation between IA and lifestyle even after adjustment (p<0.001). Nutrition and physical activity scores particularly declined for those at risk of addiction. IA was significantly associated with unhealthy dietary habits, sedentary behavior, and a lower healthy lifestyle. It may also hinder stress management and spiritual well-being. With rising internet integration, collaborative awareness programs between educational institutions, families, and communities are warranted to curb addiction risks and promote healthy lifestyles in youth. This study provides valuable insights, though longitudinal research is needed to establish causality.

## Introduction

Internet addiction (IA) is a form of digital addiction defined as a pattern of internet use that causes functional impairment and negative psychological states over a two-month period. IA has adverse impacts on individuals’ lifestyles, particularly during adolescence and young adulthood [1]. Specifically, those with IA tend to exhibit poorer lifestyle habits compared to normally functioning individuals in society [2]. According to global statistics from 2018, over 40% of the world’s population had internet access, and with the onset of the COVID-19 pandemic and the closure of in-person communication channels, internet usage and Smartphone adoption trends further increased across societies [3]. This occurred despite mounting risks of IA even as internet access grew increasingly important for educating students during the pandemic. In 2020, there were approximately 4.8 billion active internet users worldwide, accountings for 62% of the global population. IA rates in the United States in 2021 were 8.1% among 18-22year olds. Prevalence of IA across Southeast Asian countries ranged from 12.6% to 67.5% [4]. The prevalence of internet usage one year after the start of the Covid-19 pandemic among elementary and middle school students in Nepal [5], and Taiwan [6] was 21.5%, 22.8%, and 24.4%, respectively. In Iran, IA in 2022 was 31.5% [7], and IA disorders among students was 25.5% [8]. In another Iranian study, the overall prevalence of severe IA was 2.4%, which in boys was 2.9% and in girls 2%, and by age groups in adolescents was 2.5% and in youth 2.3% [9]. Adolescents are more susceptible to developing IA compared to other age groups. These statistics highlight the importance of recognizing IA as a significant health issue that demands attention among adolescents. Unregulated internet usage can increase the risk of IA among adolescents when compared to other age groups [2, 10]. The results of various studies also indicate that IA can have detrimental psychological and behavioral effects on adolescents. Some investigations have linked IA to unhealthy eating behaviors among youth such as skipping meals, low appetite, snacking between meals, inadequate nutrition, and poor diet quality [3, 11]. The prevalence of anxiety disorders has also been higher among students meeting the criteria for severe IA [3]; levels of depression and suicidal ideation were significantly greater in internet-addicted high school students in South Korea [3, 11]. IA has further reduced their levels of physical activity, and disrupted social relationships [12]. It also correlates with psychological disorders like depression, anxiety, and ineffective stress coping mechanisms. Sleep deprivation, and fatigue are common among IA [2]. Furthermore, IA is linked to other addictive behaviors such as smoking, alcohol consumption, and excessive coffee intake in young people [2, 3].

With the surge in internet adoption throughout society after the onset of the COVID-19 pandemic, these trends have significant implications for students’ lifestyles, ultimately impacting the health of the younger population [10]. However, there is still a lack of studies in Iran that focus on IA and lifestyle among adolescents following the onset of the COVID-19 pandemic. So far, the association between IA, lifestyle-related factors, and high-risk behaviors has not been clarified. Therefore, considering the recent increase in internet usage among adolescents, the aimed of this study was to examine the relationships between IA and lifestyle factors among high school students in Kurdistan province.

## Materials and methods

### Study design and population

This cross-sectional study utilized a descriptive-analytical design on high school students in Sanandaj in 2023, the capital city of Kurdistan Province in western Iran, which has a population of approximately 450,000 people. The primary aim was to examine the prevalence of IA and its relationship to lifestyle factors among high schools. Therefore, based on a previous study (p = 0.33) [13] and using a 95% confidence level, 80% power, and 5% accuracy, the sample size was calculated to be 340 using the following formula, which after applying a 10% attrition rate, the overall estimated sample was 408.


n=((Z(1−α2))^2×p(1−p)^2)/d^2=((1/96)^2×0.33(1−0.33))/((0.05)^2)=340N=n×1.2=408

### Data collection and measurements

Data was collected through cluster sampling of students based on the Ministry of Education’s three regional divisions in Sanandaj from April 2023 to September 2023. In this manner, four schools (two boys’ and two girls’ high schools) were randomly selected from each region and the estimated sample was proportionally allocated based on the number of enrolled students at each school.

The data collection tools included the Young IA Test (YIAT) [14, 15] and the Health-Promoting Lifestyle Profile (HPLP) questionnaire [16]. The 20-item YIAT uses a 5-point Likert scale ranging from ’rarely’ to ’always’ and scores from 1 to 5 respectively, with a range of 20 to 100—higher scores indicating greater IA. Scores between 20–49 indicate no IA, 50–79 indicate risk of addiction, and 80–100 indicate IA. However, given the very low prevalence of IA in this study (1.2%), those who are exposed to IA with scores above 50 were considered. The validity and reliability of this questionnaire has previously been established in Iran. The HPLP contains 36 items across six lifestyle subscales rated on a 5-point Likert scale of ’never’ to ’always’. Subscales include spiritual growth (7 items), health responsibility (8 items), nutrition (6 items), interpersonal relations (5 items), physical activity (6 items), and stress management (4 items). Scores are calculated as the mean for each dimension, ranging from 1 to 5. Validity and reliability of the Persian version of the HPLP questionnaire were evaluated by Timori et al. The Cronbach’s alpha was 82% for the whole scale [16]. Both questionnaires are standardized with established validity and reliability in Iran.

#### 1.1 Ethics approval and consent to participate

All procedures performed in studies involving human participants were approved by the Ethics Committee of the Kurdistan University of Medical Sciences under the ethics code No. IR.MUK.REC.1401.328. Also, written informed consent was obtained from all subjects.

#### 1.2. Statistical analysis

After data collection, descriptive statistics (mean, SD, frequency, percentage) and chi-square test to compare IA across groups, independent t-test to compare lifestyle dimension means between two independent groups, one-way ANOVA to compare lifestyle dimension means across other variables, and crude and adjusted linear regression analyses were utilized to examine relationships between IA as a independent variable and HPLP lifestyle factors as dependents variable while controlling for other variables. 95% confidence interval (CI) were calculated for all estimation. Based on the necessary assumptions for selecting the interlinear regression analysis method, we utilized this approach for our analysis. We assumed that the relationship between the dependent and independent variables is linear, the model adequately fits the data and has the ability to describe the relationship between the variables. Additionally, we assumed that the variables are linearly independent and that the predicted errors and residuals are independent and normally distributed (p>0.05). The Variance Inflation Factor (VIF) and forward modeling techniques revealed evidence of multicollinearity between the "Health Responsibility" and "Spiritual Growth" variables and the other predictors in the model. A variance inflation factor >5 was commiserated to represent collinearity [17]. Consequently, these two variables were excluded from the final adjusted model. Data were analyzed using SPSS software version 23.

## Results

A total of 407 students from Sanandaj city, Kurdistan province participated in the study. Female high school students comprised 55% (222 individuals) of participants, and 60% (247 individuals) were in age group of 16–19 years. 39% (161 individuals) were first grade high school students. Most participants (56%) lived in families with four or fewer family members. Their fathers and mothers had primary& intermediate (46% and 54% respectively) levels of education. Fathers were mostly self-employed (43%) while mothers were primarily homemakers (92%) (Table 1).

**Table 1:** Current status of Internet addiction based on demographic characteristics of students.

	N	Current Situation of Internet Addiction		*P-value* *
		No Internet addiction	Exposed to Internet addiction	
**Age**				
13–16 years	160	115 (71.9%)	45 (28.1%)	0.175
16–19 years	247	189 (76.5%)	58 (23.5%)	
**Sex**				
Male	185	144 (79.6%)	41 (20.4%)	0.191
Female	222	160 (74.0%)	62 (26.0%)	
**Education**				
grade 9	161	120 (74.5%)	41 (25.5%)	0.546
grade 10	136	98 (72.1%)	38 (27.9%)	
Grade 11	110	86 (78.2%)	24 (21.8%)	
**Field of study**				
Other fields	149	111 (74.4%)	38 (25.6%)	0.198
Social Sciences	87	64 (73.0%)	23 (27.0%)	
Science/ Mathematics	171	131 (76.9%)	40 (23.1%)	
**Number of family members**				
1–4 **members**	231	173 (74.9%)	58 (25.1%)	0.916
5–9 **members**	176	131 (74.4%)	45 (25.6%)	
**Father’s level of education**				
Illiterate	28	20 (71.4%)	8 (28.6%)	0.309
Primary / Middle school	191	136 (71.2%)	55 (28.8%)	
High school/Diploma	132	106 (80.3%)	26 (19.7%)	
University	56	42 (75.0%)	14 (25.0%)	
**Mother’s level of education**				
Illiterate	70	55 (78.6%)	15 (21.4%)	0.730
Primary / Middle school	223	162 (72.6%)	61 (27.4%)	
High school/Diploma	91	70 (76.9%)	21(23.1%)	
University	23	17 (73.9%)	6 (26.1%)	
**Father’s job**				
Unemployed	33	21 (63.6%)	12 (36.4%)	0.174
Laborer	107	87 (81.3%)	20 (18.7%)	
Self-employed	177	126 (71.2%)	51 (28.8%)	
Government employee	90	70 (77.8%)	20 (22.2%)	
**Mother’s job**				
Housewife	377	283 (75.1%)	94 (24.9%)	0.539
Employed	30	21 (70.0%)	9 (30.0%)	
**Participate in Language/Sport/ Art Class**				
Yes	203	157 (51.6%)	46 (44.7%)	0.133
No	204	147 (48.6%)	57 (55.3%)	
**Total**	407	304 (74.7%)	103 (25.3%)	

*by chi-square test

Overall, as shown in Table 1, 25% (103 individuals) of students were exposed to IA. This was higher among female high school students (26%), those aged 16 and younger (28%), second grade high school students (28%), humanities students (27%), families where fathers and mothers had primary/intermediate education levels (29–27%), and students with unemployed fathers (36%) or employed mothers (30%). However, none of these percentages differed significantly statistically (p>0.05) (Table 1).

Based on Table 2, the following results can be extracted: The overall score of the health-promoting lifestyle was 120.3±26.0, with the highest score observed among individuals who were not exposed to IA, scoring 126.0±24.2, showing a statistically significant difference compared to those were exposed to IA. Furthermore, individuals who did not participate in miscellaneous classes had the highest statistically significant score of 124.7±25.3. Finally, the highest score of 123.3±26.9 was associated with individuals from families with a population of more than 4 members (p<0.05). Additionally, in Table 2, Spiritual Growth was higher among students over 16 years old and in families with less educated fathers. Health Responsibility and Nutrition were lower among students who attended remedial classes and had IA. Interpersonal Relations were higher among families with larger populations, lower-educated fathers, and individuals who were not exposed to IA. Physical Activity was also higher among males, families with larger populations, students who did not attend remedial classes, and those who were not exposed to internet addiction. Finally, Physical Activity was reported less among students who were exposed to internet addiction (p<0.05).

**Table 2:** Current status of health-promoting lifestyle based on demographic characteristics of students.

	Total (36 items)	Health-Promoting Lifestyle					
		Spiritual Growth (7 items)	Health Responsibility (8 items)	Nutrition (6 items)	Interpersonal Relations (5 items)	Physical Activity (6 items)	Stress Management (4 items)
**Age**							
16≥	118.8±25.9	***27*.*8±5*.*4***	23.0±7.8	20.56±5.0	15.83±4.9	17.39±6.4	14.13±4.1
>16	121.2±26.0	***28*.*9±5*.*4***	23.62±8.0	20.64±4.9	16.16±5.1	17.37±6.6	14.51±3.6
**Sex**							
Male	125.6±24.0	29.0±5.2	23.2±7.1	21.6±4.7	16.9±4.6	***20*.*2±5*.*5***	14.5±3.8
Female	119.6±26.2	28.4±5.4	23.4±8.0	20.4±4.9	15.9±5.1	***16*.*9±6*.*5***	14.3±3.7
**Education**							
First high school	118.6±26.4	27.8±5.7	23.0±7.9	20.6±5.0	15.7±5.0	17.3±6.5	14.0±4.0
2nd high school	121.7±25.6	29.1±4.7	23.4±8.1	20.4±5.0	16.3±5.1	17.7±6.7	14.5±3.7
3rd high school	121.0±25.8	28.8±5.6	23.8±7.9	20.8±4.8	15.9±5.0	17.0±6.3	14.5±3.3
**Field of study**							
Other fields	120.3±26.9	28.3±5.6	23.7±8.1	20.2±4.9	16.2±4.9	17.3±6.6	14.3±3.8
Social Sciences	121.0±25.2	28.8±5.1	23.3±7.8	20.7±4.9	15.6±5.2	17.8±6.5	14.6±3.5
Science/ Mathematics	119.4±24.2	28.6±5.0	22.5±7.5	21.5±4.8	15.7±5.0	17.0±6.2	14.0±3.9
**Number of family members**							
4≥	***118*.*0±25*.*0***	28.2±5.3	23.0±7.3	20.4±4.8	***15*.*4±4*.*9***	***16*.*7±6*.*5***	14.1±3.7
>4	***123*.*3±26*.*9***	28.9±5.5	23.9±8.7	20.8±5.1	***16*.*7±5*.*1***	***18*.*1±6*.*4***	14.6±3.8
**Father’s level of education**							
Illiterate	123.5±23.5	***30*.*6±4*.*4***	24.2±6.7	19.9±4.7	***18*.*2±4*.*3***	16.1±7.1	14.2±3.5
Primary / Middle school	118.7±26.1	***28*.*0±5*.*5***	23.3±8.1	20.4±4.9	***15*.*8±5*.*1***	16.9±6.2	14.1±3.8
High school/Diploma	123.8±26.3	***29*.*1±5*.*2***	23.7±8.1	21.1±5.1	***16*.*5±4*.*8***	18.4±6.6	14.8±3.6
University	115.6±25.2	***27*.*6±5*.*5***	22.5±7.6	20.3±4.7	***14*.*3±5*.*0***	17.1±6.9	13.7±3.7
**Mother’s level of education**							
Illiterate	119.4±27.8	28.0±5.9	23.7±8.2	20.1±5.3	16.9±4.9	16.2±6.5	14.2±4.3
Primary / Middle school	120.8±25.9	28.6±5.2	23.5±8.0	20.7±4.7	15.7±5.1	17.6±6.2	14.4±3.6
High school/Diploma	120.0±24.1	28.7±5.1	22.9±7.8	20.6±5.0	16.3±4.6	17.1±7.0	14.3±3.5
University	119.3±29.2	28.3±6.2	22.8±7.3	20.2±5.7	14.7±5.4	19.5±6.7	13.6±4.8
**Father’s job**							
Unemployed	109.0±27.3	120.±26.	29.4±5.8	24.1±8.2	20.5±4.8	16.6±4.7	14.9±6.5
Worker	122.2±25.3	27.4±5.9	28.5±5.4	25.7±9.4	20.5±5.0	16.1±4.8	17.4±6.0
Market jobs	119.6±24.4	28.6±5.4	20.9±7.4	23.4±7.9	21.5±4.8	15.4±5.4	17.2±6.5
Employee	121.6±26.0	28.3±5.3	23.9±7.7	18.7±5.6	20.6±4.9	16.3±5.6	18.1±6.9
**Mother’s job**							
Housewife	120.5±26.0	28.5±5.4	23.5±8.0	20.6±4.9	16.1±5.0	17.3±6.5	14.4±3.7
Employed	117.6±25.1	29.1±4.5	22.1±7.4	20.0±5.3	14.6±5.5	17.9±7.1	13.8±3.8
**Participate in Language/Sport/ Art Class**							
Yes	***115*.*9±25*.*9***	***27*.*8±5*.*7***	***22*.*1±8*.*0***	***19*.*9±5*.*0***	15.9±4.9	***15*.*6±5*.*9***	14.2±3.9
No	***124*.*7±25*.*3***	***29*.*1±5*.*0***	***24*.*6±7*.*7***	***21*.*2±4*.*8***	16.1±5.1	***19*.*0±6*.*6***	14.4±3.6
**Current Situation of Internet Addiction**							
**No Internet addiction**	***126*.*0±24*.*2***	***29*.*7±4*.*5***	***25±7*.*6***	***21*.*4±4*.*6***	***16*.*7±4*.*8***	***18*.*2±6*.*3***	***14*.*9±3*.*4***
**Exposed to Internet addiction**	***103*.*4±23*.*7***	***25*.*0±6*.*2***	***18*.*7±7*.*1***	***18*.*2±5*.*1***	***14*.*0±5*.*0***	***14*.*8±6*.*4***	***12*.*5±4*.*0***
**Total**	120.3±26.0	28.54±5.42	23.42±7.98	20.61±4.97	16.03±5.05	17.38±6.54	14.36±3.79

*bold: P<0.05 by independent t-test and one-way ANOVA

Data are presented as mean ± SD

Table 3 illustrates the relationship between internet addiction and Health-Promoting lifestyle among high school students using two models: a single-variable model and an adjusted model. In the single-variable model, a significant relationship was observed between internet addiction and Health-Promoting lifestyle, whereby those exposed to internet addiction had lower Health-Promoting Lifestyle scores (ß = -22.6, P < 0.001). This inverse association was also present for other components (P < 0.05). In the adjusted model, even after controlling for other variables, there remained a significant inverse association between internet addiction and Health-Promoting Lifestyle. Specifically, the Health-Promoting Lifestyle score was 22.2 units lower (95% CI: 27.5, -16.9, P < 0.001) among those exposed to internet addiction. Similar statistically significant reductions were observed for the Nutrition and Physical Activity components (P < 0.05).

**Table 3:** Linear regression analysis to assess association between internet addiction and lifestyle among high school students.

	Spiritual Growth	Health Responsibility	Nutrition	Interpersonal Relations	Physical Activity	Stress Management	Health-Promoting Lifestyle
***Exposed to Internet addiction (No is Reference)***							
**Crude**							
***ß* (95% CI)**	-4.72 (-5.8, -3.6)	-6.2 (-7.9, -4.57)	-3.1 (-4.2, -2.1)	-2.6 (-3.7,-1.5)	-3.3 (-4.7,-1.92)	-2.4 (-3.2, -1.6)	-22.6 (-28.0,-17.2)
**P value** *	***<0*.*001***	***<0*.*001***	***<0*.*001***	***<0*.*001***	***<0*.*001***	***<0*.*001***	***<0*.*001***
**Adjust**							
***ß* (95% CI)**	…	…	-0.94 (-1.8,-0.03)	-0.99 (-2.0,0.03)	-0.61 (-1.8,0.59)	-.69 (-1.3,-0.01)	-22.2 (-27.5,-16.9)
**P value**	…	…	***0*.*043***	0.057	***0*.*046***	0.315	***<0*.*001***

* Crude and adjusted linear regression to examine relationships between Internet addiction as an independent variable and HPLP lifestyle factors as dependent variables. Exposed to Internet addiction is an independent variable (and HPLP lifestyle factors are dependents variables.

Crude: a single-variable model

Adjust: controlling for other variable (Age, Sex, Education, Field of study, Number of family members, Mother’s level of education, Father’s job, Mother’s job and Participate in Language/Sport/ Art Class)

## Discussion

This research investigates the correlation between IA and Health-Promoting Lifestyle among high school students in Kurdistan Province. The study indicates that approximately 25% of high school students are vulnerable to IA. Similar patterns of IA prevalence have been observed in other regions, such as Isfahan, where 58.8% of high school students were susceptible to and addicted to the internet [18]. Among medical students, the figure was reported to be 68.8% [19]. The widespread availability and affordability of internet services, along with the increasing use of the internet for educational and recreational purposes, as well as the extensive adoption of smartphones, contribute to the rising prevalence of IA among young individuals [20]. Furthermore, this trend is influenced by increased individualism, decreased social acceptance, and cultural adaptability [3].

It is important to note that the data presented in this study predates the COVID-19 pandemic, and, likely, the prevalence of IA has significantly increased in light of the circumstances. Recent reports from Brazil indicate that over half of the students suffer from IA problems [21]. Similarly, studies conducted during the early stages of the pandemic in 2019 revealed high rates of IA among Tunisian adolescents (43.9%) [22], Mexican students (62.7%) [23], medical and dental students in Pakistan(40.9%) [11], and Ecuadorian students in 2022 (51.0%) [24]. It is worth mentioning that in the current study, less than 2% of participants exhibited complete addiction to the internet, while 25% were exposed to IA overall. However, it is important to consider that variations in these statistics may arise due to differences in socio-demographic contexts or assessment methods for IA.

In recent years, there have been significant changes in individuals’ lives, leading to the prevalence of unhealthy lifestyle choices such as poor nutrition, unhealthy diet, smoking, alcohol consumption, substance abuse, and stress [25]. This study, consistent with previous research [26, 27], establishes a substantial inverse association between IA and a healthy lifestyle. The findings indicate that individuals at risk of IA scored 22.2 points lower on the healthy lifestyle scale. Previous studies have demonstrated that IA is linked to reduced physical activity, negative effects on nutrition, and disruptions in social relationships. Additionally, it is associated with psychological disorders such as depression, anxiety, and diminished stress coping mechanisms [28–30]. Some studies reported that IA is correlated with sleep deprivation, symptoms of fatigue (both physical and mental), and frequent consumption of fast food [31, 32]. Excessive internet use appears to be an unhealthy lifestyle choice for individuals addicted to the internet. Researches have also shown a correlation between IA and other addictive behaviors such as smoking, alcohol consumption, and excessive coffee consumption among young people [2, 33]. Therefore, IA can have far-reaching negative effects on individuals’ lifestyles and well-being.

While studies have documented a rising trend of low physical activity among Iranian and global adolescents [12]. IA has further reduced their levels of physical activity [2, 30, 34]. Similar to the present study, research conducted in Malaysia found a strong connection between IA and sedentary behavior (defined as more than three hours of sitting per day) among adolescents [2]. Internet-addicted adolescents often spend extended periods sitting in front of a screen. Internet addiction may lead to decreased physical activity and indirectly contribute to obesity an studies have reported higher body mass index values in individuals with problematic internet use compared to those with normal internet use [2, 35]. Moreover, research indicates that individuals spend their leisure time using the internet and engaging in sedentary behaviors, displaying little inclination for physical activity. For many university students, low-intensity activities such as slow walking have become their primary form of physical activity [36, 37].

Although studies have highlighted inadequate nutrition trends, low nutritional literacy, and poor nutrition among Iranian students [38, 39], the current study also reveals a significant inverse relationship between internet addiction and nutrition scores. A study among high school students demonstrated that high-risk internet users exhibited inappropriate dietary behavior and poor dietary quality. These individuals consumed smaller meals, had reduced appetite, skipped meals, and snacked more frequently [11, 40]. Bakery products and high-calorie foods were popular snack choices among participants, and a significant association was found between meat and fried food consumption and IA [11]. The study also found that unhealthy dietary behaviors such as insufficient intake of fruits and vegetables, excessive consumption of carbonated beverages, and frequent fast-food consumption were positively associated with IA among adolescents. These findings align with a study, which reported a high prevalence of meal skipping among individuals addicted to the internet [40]. Conversely, healthy dietary behaviors such as regular breakfast consumption and adequate intake of fruits and vegetables have been shown to be beneficial for emotional regulation [41] and reducing negative emotions in adolescents [2, 42]. According to a systematic review [43], adolescents who exhibit healthy dietary behaviors or consume high-quality diets experience lower levels of depression and better psychological well-being.

This study investigates the association between IA and various dimensions of a health-promoting lifestyle. The results of the single-variable regression analysis revealed a negative correlation between IA and different dimensions of a health-promoting lifestyle. These findings are consistent with previous research findings. For instance, a similar study found that IA has a detrimental impact on spiritual growth [44], and religiosity serves as a protective factor against IA [45]. Moreover, individuals who excessively use the Internet or lack self-control over their Internet usage tend to exhibit lower health responsibilities [46]. It has also been observed that adolescents who use the Internet safely demonstrate better social skills compared to those who do not use it safely [47]. Furthermore, a study has indicated that individuals with IA experience significantly poorer stress management compared to non-addicted individuals [48]. Therefore, IA significantly influences a health-promoting lifestyle.

### Limitation

However, this study has certain limitations, including its cross-sectional design, which restricts the establishment of causal relationships. Additionally, reliance on self-reported measures for IA and lifestyle behaviors introduces the possibility of response bias. Furthermore, the study’s sample size may also be considered a limitation. With a larger and more diverse sample, we would have had a greater representation of the population, allowing for more generalizable findings.

### Strength

The article demonstrates notable strengths in its methodology and analysis. It utilizes standardized and validated measurement tools, such as the Young Internet Addiction Test and the Health-Promoting Lifestyle Profile, to assess IA and health-promoting lifestyle, respectively. Additionally, the use of regression analysis allows for an examination of the relationship between IA and health-promoting lifestyle while controlling for other variables. These methodological choices enhance the credibility of the results and facilitate meaningful insights.

## Conclusion

This study examined the association between IA and lifestyle among high school students in the west of Iran. The findings revealed that around 25% of the participants were exposed to IA. Students weren’t exposed to IA demonstrated significantly higher scores in overall healthy lifestyle. Moreover, significant differences were observed in the areas of spiritual growth, health responsibility, and nutrition between the two groups. Regression analyses confirmed a negative correlation between exposed to IA and lifestyle, even after accounting for other variables. Specifically, individuals exposed to IA exhibited declines in nutrition and physical activity scores. IA was found to be significantly linked to unhealthy dietary habits, sedentary behavior, and a lower overall healthy lifestyle. Additionally, it hindered stress management and spiritual well-being. This study highlights the importance of collaborative awareness programs involving educational institutions, families, and communities to address the risks of addiction and promote healthy lifestyles among young individuals. While providing valuable insights, further longitudinal research is needed to establish causal relationships definitively.

## References

[1] Marzilli E, Cerniglia L, Ballarotto G, Cimino S. Internet addiction among young adult university students: The complex interplay between family functioning, impulsivity, depression, and anxiety. International journal of environmental research and public health. 2020;17(21):8231. doi: 10.3390/ijerph17218231 33171742 PMC7664422

[2] Ying Ying C, Awaluddin SM, Kuang Kuay L, Siew Man C, Baharudin A, Miaw Yn L, et al. Association of internet addiction with adolescents’ lifestyle: a national school-based survey. International journal of environmental research and public health. 2020;18(1):168. doi: 10.3390/ijerph18010168 33383635 PMC7801949

[3] Lozano-Blasco R, Robres AQ, Sánchez AS. Internet addiction in young adults: A meta-analysis and systematic review. Computers in Human Behavior. 2022;130:107201.

[4] Kuss DJ, Kristensen AM, Lopez-Fernandez O. Internet addictions outside of Europe: A systematic literature review. Computers in Human Behavior. 2021;115:106621.

[5] Karki K, Singh DR, Maharjan D, KC S, Shrestha S, Thapa DK. Internet addiction and sleep quality among adolescents in a peri-urban setting in Nepal: A cross-sectional school-based survey. PLoS One. 2021;16(2):e0246940. doi: 10.1371/journal.pone.0246940 33600410 PMC7891762

[6] Lin M-P. Prevalence of internet addiction during the COVID-19 outbreak and its risk factors among junior high school students in Taiwan. International journal of environmental research and public health. 2020;17(22):8547. doi: 10.3390/ijerph17228547 33218018 PMC7698622

[7] Salarvand S, N. Albatineh A, Dalvand S, Baghban Karimi E, Ghanei Gheshlagh R. Prevalence of internet addiction among iranian university students: a systematic review and meta-analysis. Cyberpsychology, Behavior, and Social Networking. 2022;25(4):213–22. doi: 10.1089/cyber.2021.0120 35085012

[8] Sarikhani Y, Rafiee M, Ostovar T. The Association of Internet Addiction Disorder with Personality Type and Parenting Style among High School Students in Jahrom, Southern Iran. International Journal of School Health. 2021;8(4):241–6.

[9] Mousavi SV-a. Prevalence of Internet addiction and the Status of the use of virtual social networks in Iranian Teenagers and Youths in 2018. Mil Med. 2020;22(3):281–8.

[10] Çelik DÖ, Haney MÖ. The relationship between depression, healthy lifestyle behaviors and internet addiction: a cross-sectional study of the athlete university students in Turkey. Frontiers in Psychiatry. 2023;14:1222931. doi: 10.3389/fpsyt.2023.1222931 37484666 PMC10359973

[11] Waheed W, Jamil W, Rahat T, Zahra S, Perwaiz M, Amjad S, Raza Q. Relationship between Internet Addiction and Dietary Behaviors of Students, Studying in a Teaching Hospital. International Journal of Nutrition Sciences. 2021;6(4):189–93.

[12] Amiri P, Naseri P, Vahedi-Notash G, Jalali-Farahani S, Mehrabi Y, Hamzavi-Zarghani N, et al. Trends of low physical activity among Iranian adolescents across urban and rural areas during 2006–2011. Scientific reports. 2020;10(1):21318. doi: 10.1038/s41598-020-78048-0 33288806 PMC7721745

[13] Rastegarian A, Bazrafshan A, Keshavarz P. Happiness and Internet Addiction among High School Girls in Iran: A Single-Center Experience. Iranian Journal of Psychiatry. 2022;17(4):388. doi: 10.18502/ijps.v17i4.10687 36817814 PMC9922348

[14] Ghassemzadeh L, Shahraray M, Moradi A. Prevalence of Internet addiction and comparison of Internet addicts and non-addicts in Iranian high schools. CyberPsychology & Behavior. 2008;11(6):731–3. doi: 10.1089/cpb.2007.0243 18954277

[15] Young KS. Caught in the net: How to recognize the signs of internet addiction—and a winning strategy for recovery: John Wiley & Sons; 1998.

[16] Taymoori P, Moeini B, Lubans D, Bharami M. Development and psychometric testing of the Adolescent Healthy Lifestyle Questionnaire. Journal of education and health promotion. 2012;1. doi: 10.4103/2277-9531.99221 23555123 PMC3577412

[17] Akinwande MO, Dikko HG, Samson A. Variance inflation factor: as a condition for the inclusion of suppressor variable (s) in regression analysis. Open journal of statistics. 2015;5(07):754.

[18] Fathian Dastgerdi Z, Amidi Mazaheri M, Jadidi H, Zhaleh M, Kaviani Tehrani A, Ghasemi M. Prevalence of internet addiction and its association with general health status among high school students in Isfahan, Iran. International Journal of Pediatrics-Mashhad. 2020;8(1):10799–806.

[19] Khazaie H, Lebni JY, Abbas J, Mahaki B, Chaboksavar F, Kianipour N, et al. Internet addiction status and related factors among medical students: a cross-sectional study in Western Iran. Community Health Equity Research & Policy. 2023;43(4):347–56. doi: 10.1177/0272684X211025438 34128427

[20] Mohanty R, Dey P, Hebbar N, Singh H. Effect of internet use on medical students before and after 4 g internet service in india: A comparative study. L’encephale. 2021;47(3):189–94. doi: 10.1016/j.encep.2020.10.001 33309007

[21] Brito AB, Lima CdA, Brito KDP, Freire RS, Messias RB, Rezende LFd, et al. Prevalence of internet addiction and associated factors in students. Estudos de Psicologia (Campinas). 2023;40:e200242.

[22] Thabet JB, Ellouze A, Ghorbel N, Maalej M, Yaich S, Omri S, et al. Facteurs associés à la cyberaddiction chez les adolescents Tunisiens. L’Encéphale. 2019;45(6):474–81. 10.1016/j.encep.2019.05.006 31421811

[23] Priego-Parra BA, Triana-Romero A, Pinto-Gálvez SM, Ramos CD, Salas-Nolasco O, Reyes MM, et al. Anxiety, depression, attitudes, and internet addiction during the initial phase of the 2019 coronavirus disease (COVID-19) epidemic: A cross-sectional study in México. MedRxiv. 2020:2020.05.10.20095844.

[24] Jiménez SOI, Jimenez IJI, Pierard SMC, Jiménez ACI. Relación entre uso problemático de internet y calidad de sueño durante la pandemia de COVID-19. Universidad Ciencia y Tecnología. 2021;25(109):116–23.

[25] Kapus K, Nyulas R, Nemeskeri Z, Zadori I, Muity G, Kiss J, et al. Prevalence and risk factors of internet addiction among hungarian high school students. International Journal of Environmental Research and Public Health. 2021;18(13):6989. doi: 10.3390/ijerph18136989 34208800 PMC8297371

[26] Kaya A, Dalgiç AI. How does internet addiction affect adolescent lifestyles? Results from a school-based study in the Mediterranean region of Turkey. Journal of pediatric nursing. 2021;59:e38–e43. doi: 10.1016/j.pedn.2021.01.021 33589290

[27] Gülbetekin E, Güven E, Sökmen Ö. The effect of internet addiction on physical activity and dietary habits in high school students. Journal of Public Health. 2023:1–8.

[28] Oluwole LO, Obadeji A, Dada MU. Surfing over masked distress: Internet addiction and psychological well-being among a population of medical students. Asian Journal of social health and behavior. 2021;4(2):56–61.

[29] İbrahim T. Association between depression, anxiety, stress, social support, resilience and internet addiction: A structural equation modelling. Malaysian Online Journal of Educational Technology. 2019;7(3):1–10.

[30] Gałczyk M, Zalewska A, Białokoz-Kalinowska I, Sobolewski M. Chronic back condition and the level of physical activity as well as internet addiction among physiotherapy students during the COVID-19 pandemic in Poland. International Journal of Environmental Research and Public Health. 2021;18(13):6718. doi: 10.3390/ijerph18136718 34206426 PMC8297369

[31] Alimoradi Z, Lin C-Y, Broström A, Bülow PH, Bajalan Z, Griffiths MD, et al. Internet addiction and sleep problems: A systematic review and meta-analysis. Sleep medicine reviews. 2019;47:51–61. doi: 10.1016/j.smrv.2019.06.004 31336284

[32] Bener A, Yildirim E, Torun P, Çatan F, Bolat E, Alıç S, et al. Internet addiction, fatigue, and sleep problems among adolescent students: A large-scale study. International Journal of Mental Health and Addiction. 2019;17:959–69.

[33] Lazea C, Popa A, Varga C. Association between internet use behavior and palpitation among adolescents: A cross-sectional study of Middle school children from Northwest Romania. International Journal of Environmental Research and Public Health. 2020;17(12):4278. doi: 10.3390/ijerph17124278 32549329 PMC7344809

[34] Kwok C, Leung PY, Poon KY, Fung XC. The effects of internet gaming and social media use on physical activity, sleep, quality of life, and academic performance among university students in Hong Kong: A preliminary study. Asian Journal of Social Health and Behavior. 2021;4(1):36–44.

[35] Bozkurt H, Özer S, Şahin S, Sönmezgöz E. Internet use patterns and Internet addiction in children and adolescents with obesity. Pediatric obesity. 2018;13(5):301–6. doi: 10.1111/ijpo.12216 28371539

[36] Cheng M, Wang S, Wang Y, Zhang R, Qin L. Physical activity reduces internet addiction among “post-00” college students: The mediating role of coping styles. Frontiers in Psychology. 2023;13:1052510. doi: 10.3389/fpsyg.2022.1052510 36846475 PMC9945914

[37] Gülşah K, Soyocak A, Ongün P, KERVANCIOĞLU G. Assessment of eating habits and internet addiction levels based on the physical activity levels in university students. Düzce Üniversitesi Sağlık Bilimleri Enstitüsü Dergisi. 2021;11(1):25–32.

[38] Kooshki A, Mohammadi M, Rivandi M. Nutritional intake and its association with educational achievement in high-school students in Islamic Republic of Iran. Eastern Mediterranean Health Journal. 2018;24(6):532–7. doi: 10.26719/2018.24.6.532 30079948

[39] Ashoori M, Omidvar N, Eini-Zinab H, Shakibazadeh E, Doustmohamadian A, Abdar-Esfahani B, Mazandaranian M. Food and nutrition literacy status and its correlates in Iranian senior high-school students. BMC nutrition. 2021;7:1–10. 34082827 10.1186/s40795-021-00426-2 PMC8176697

[40] Mekhemer FA, Ahmed ES, Hassan AM. Consequences of internet addiction on dietary behavior among adolescents in Assiut City. Assiut Scientific Nursing Journal. 2019;7(17):13–23.

[41] Isasi CR, Ostrovsky NW, Wills TA. The association of emotion regulation with lifestyle behaviors in inner-city adolescents. Eating behaviors. 2013;14(4):518–21. doi: 10.1016/j.eatbeh.2013.07.009 24183148 PMC3817414

[42] Yao L, Liang K, Huang L, Chi X. Relationship between fruit and vegetable consumption and internet addiction with insomnia and depression as multiple mediators during the COVID-19 pandemic: a three-wave longitudinal study in Chinese college students. BMC psychiatry. 2023;23(1):1–11. 38093234 10.1186/s12888-023-05415-2 PMC10720225

[43] Khalid S, Williams CM, Reynolds SA. Is there an association between diet and depression in children and adolescents? A systematic review. British journal of nutrition. 2016;116(12):2097–108. doi: 10.1017/S0007114516004359 28093091

[44] Çelik DÖ, Haney MÖ. The relationship between depression, healthy lifestyle behaviors and internet addiction: a cross-sectional study of the athlete university students in Turkey. Frontiers in Psychiatry. 2023;14. doi: 10.3389/fpsyt.2023.1222931 37484666 PMC10359973

[45] Dossi F, Buja A, Montecchio L. Association between religiosity or spirituality and internet addiction: A systematic review. Frontiers in Public Health. 2022;10:980334. doi: 10.3389/fpubh.2022.980334 36530734 PMC9751319

[46] Lin P-H, Lee Y-C, Chen K-L, Hsieh P-L, Yang S-Y, Lin Y-L. The relationship between sleep quality and internet addiction among female college students. Frontiers in neuroscience. 2019;13:599. doi: 10.3389/fnins.2019.00599 31249504 PMC6582255

[47] GÜNDOĞDU NA, TOSUN AS, YILDIZ İ, MERT ZT. The Effect of The Internet on Adolescents: A Mixed-Method Study. Turkish Journal of Family Medicine and Primary Care. 2022;16(4):711–24.

[48] Saidi NA, Ab Ghaffar SF, Yee Sa C, Ezani NN, Zhani A, Shuhada N, et al. Relationship between stress and lifestyle with internet addiction among Z generation. Journal of Tourism, Hospitality & Culinary Arts (JTHCA). 2022.

